# The CT-based intratumoral and peritumoral machine learning radiomics analysis in predicting lymph node metastasis in rectal carcinoma

**DOI:** 10.1186/s12876-022-02525-1

**Published:** 2022-11-16

**Authors:** Hang Yuan, Xiren Xu, Shiliang Tu, Bingchen Chen, Yuguo Wei, Yanqing Ma

**Affiliations:** 1 General Surgery, Cancer Center, Department of Colorectal Surgery, Zhejiang Provincial People’s Hospital, Hangzhou Medical College, Hangzhou, 310014 Zhejiang China; 2 Cancer Center, Department of Radiology, Zhejiang Provincial People’s Hospital, Hangzhou Medical College, Hangzhou, 310014 Zhejiang China; 3 GE Healthcare, Precision Health Institution, Hangzhou, Zhejiang China

**Keywords:** Rectal carcinoma, Lymph node metastasis, Radiomics, Intratumoral, Peritumoral

## Abstract

**Background:**

To construct clinical and machine learning nomogram for predicting the lymph node metastasis (LNM) status of rectal carcinoma (RC) based on radiomics and clinical characteristics.

**Methods:**

788 RC patients were enrolled from January 2015 to January 2021, including 303 RCs with LNM and 485 RCs without LNM. The radiomics features were calculated and selected with the methods of variance, correlation analysis, and gradient boosting decision tree. After feature selection, the machine learning algorithms of Bayes, k-nearest neighbor (KNN), logistic regression (LR), support vector machine (SVM), and decision tree (DT) were used to construct prediction models. The clinical characteristics combined with intratumoral and peritumoral radiomics was taken to develop a radiomics and machine learning nomogram. The relative standard deviation (RSD) was used to predict the stability of machine learning algorithms. The area under curves (AUCs) with 95% confidence interval (CI) were calculated to evaluate the predictive efficacy of all models.

**Results:**

To intratumoral radiomics analysis, the RSD of Bayes was minimal compared with other four machine learning algorithms. The AUCs of arterial-phase based intratumoral Bayes model (0.626 and 0.627) were higher than these of unenhanced-phase and venous-phase ones in both the training and validation group.The AUCs of intratumoral and peritumoral Bayes model were 0.656 in the training group and were 0.638 in the validation group, and the relevant Bayes-score was quantified. The clinical-Bayes nomogram containing significant clinical variables of diameter, PNI, EMVI, CEA, and CA19-9, and Bayes-score was constructed. The AUC (95%CI), specificity, and sensitivity of this nomogram was 0.828 (95%CI, 0.800-0.854), 74.85%, and 77.23%.

**Conclusion:**

Intratumoral and peritumoral radiomics can help predict the LNM status of RCs. The machine learning algorithm of Bayes in arterial-phase conducted better in consideration of terms of RSD and AUC. The clinical-Bayes nomogram achieved a better performance in predicting the LNM status of RCs.

**Supplementary information:**

The online version contains supplementary material available at 10.1186/s12876-022-02525-1.

## Introduction

Rectal carcinoma (RC) is one of the leading causes of cancer related death, accounting for nearly 43.4% of all new colorectal carcinomas diagnosed in 2021 [1]. The 5-year survival rates of patients with RC varied widely ranging from 59.1 to 70.9% in seven high-income countries between 2010 and 2014, according to their different heterogeneity [2]. Its pathological features of lymphovascular invasion have been reported to guide the individual treatment and prognostication [3]. It has been reported that approximately 10% of T1 colorectal carcinoma occurred lymph node metastasis (LNM), possibly increasing the risk of positive surgical margin and associated postoperative mortality [4]. The preoperative evaluation of LNM can provide important information to determine the necessity for adjuvant therapy and the appropriateness of surgeries [5]. CT is the most frequently used radiological techniques in evaluating the clinical staging and guiding the therapy, but lacking of consensus on a standard definition of LNM limited its diagnostic accuracy [6]. Therefore, improving the approach to preoperatively identify the high risk status of lymph node in RC patients, and therefore improving treatment targeting, is of great importance [7].

Radiomics is a computer-aid technique for high-throughput mining of quantitative image features from conventional radiological images that allows data to be applied in clinical decision, is gaining increasing attention [8]. It has been reported that T2-weighted and apparent diffusion coefficient based MRI radiomics combined with clinical data can improve efficacy in predicting the status of LNM [9]. And the high-resolution MRI-based radiomic nomogram showed good predictive performance in predicting the LNM of RC, preoperatively [10]. The radiomics and deep learning models also performed better than radiologists to predict LNM in rectal carcinoma [11]. The dual-energy CT radiomics evaluated the largest short-axis lymph node found that it can help predict the LNM in RC [12]. While, to best of our knowledge, the routine CT-based intratumoral and peritumoral radiomics analysis to assess the LNM status of RC has been neglected. The purpose of this article is to predict the LNM status in RC via a machine learning approach to analyze CT-based intratumoral and peritumoral radiomics.

## Methods and materials

### Patients enrollment

This retrospective study was approved by the Medical ethics committee of our hospital (No. 2021QT339) and the informed consent of patients was waived. After searching the surgical database of our hospital, a cohort of 788 patients which were histopathologically diagnosed as rectal carcinoma were enrolled in this study from January 2015 to January 2021. The specific inclusion criteria were lesions which were happened in rectum or the junction between rectum and sigmoid colon, were histopathologically diagnosed as classical adenocarcinoma, signet-ring cell carcinoma, or mucinous carcinoma, were taken triphasic CT examinations, and received surgeries within two weeks after CT examinations. The exclusion criteria were patients who had a history of metachronous or recurrent malignancy, received chemotherapy or radiation therapy before surgeries, and were happened in the ascending, descending, or sigmoid colon.

**Fig. 1:**
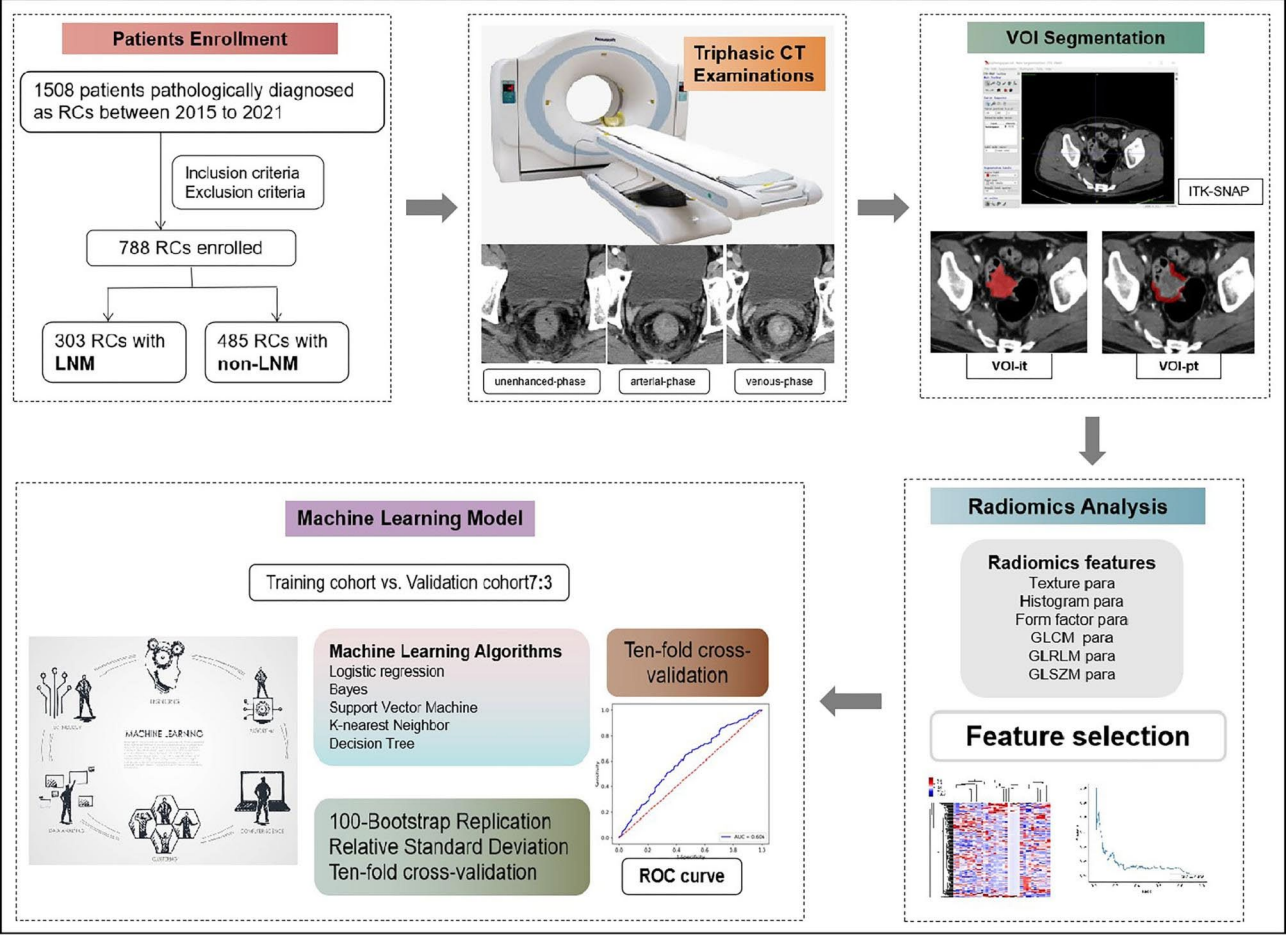
The general technical workflow of this study

The general technical workflow was illustrated in Fig. 1. Finally, the cohort including 303 RCs with LNM and 485 RCs without LNM (non-LNM) was randomly divided into the training group (212 LNM and 339 non-LNM) and validation group (91 LNM and 146 non-LNM) with a proportion of 7:3.

### CT examination

All patients underwent triphasic CT examinations with a 64 or 128 slices CT protocol (Somatom Definition AS, Siemens, Germany) with the same parameters: tube voltage 120Kv, tube current 200mA, collimation 64*0.625, field of view 360 mm, rotation time 0.75s, slice and thickness interval 5 mm. The triphasic CT examination including unenhanced-phase, arterial-phase, and venous-phase were carried out by the method of computer-aid bolus tracking (1.3 mL/Kg iomeperol 350, 3.0 mL/s) by injecting contrast media via elbow vein. After a delay of 35 and 60 s of unenhanced phase, the arterial phase and venous phase were performed, respectively.

### Clinical characteristics

The histopathological characteristics of LNM was diagnosed according to the American Joint Commission on Cancer TNM staging system and the ESMO Clinical Practice Guideline for diagnosis of colon cancer [13]. When the number of positive regional lymph node greater than or equal to one was regarded as LNM, otherwise the absence of positive regional lymph node was classified into non-LNM. The clinical characteristics included gender, age, long diameter, location (It was divided into low, medium, and high position according to the lesion distance within 5 cm, between 5 and 10 cm, and higher than 10 cm from the anal margin), perineural invasion (PNI), extramural venous invasion (EMVI), microsatellite instability (MSI), carcinoembryonic antigen (CEA), carbohydrate antigen 19 − 9 (CA19-9), history of diabetes, hypertension, smoking, and drinking. Additionally, the tumor located at the recto-sigmoid region and more than 10 cm away from the anal margin was classified as high RC. The PNI refers to a process of neoplastic invasion of nerves, nerve sheaths, and the surrounding tissues, which is recognized as a route of metastatic spread [14]. The presence of EMVI was defined as the involvement of tumor to the vasculature beyond the muscularis propria [15]. Tumors lacked one or more mismatch repair proteins of MLH1, MSH2, MSH6, and PMS2 were expected to be MSI status [16].

### CT-based machine learning radiomics analysis

**Fig. 2:**
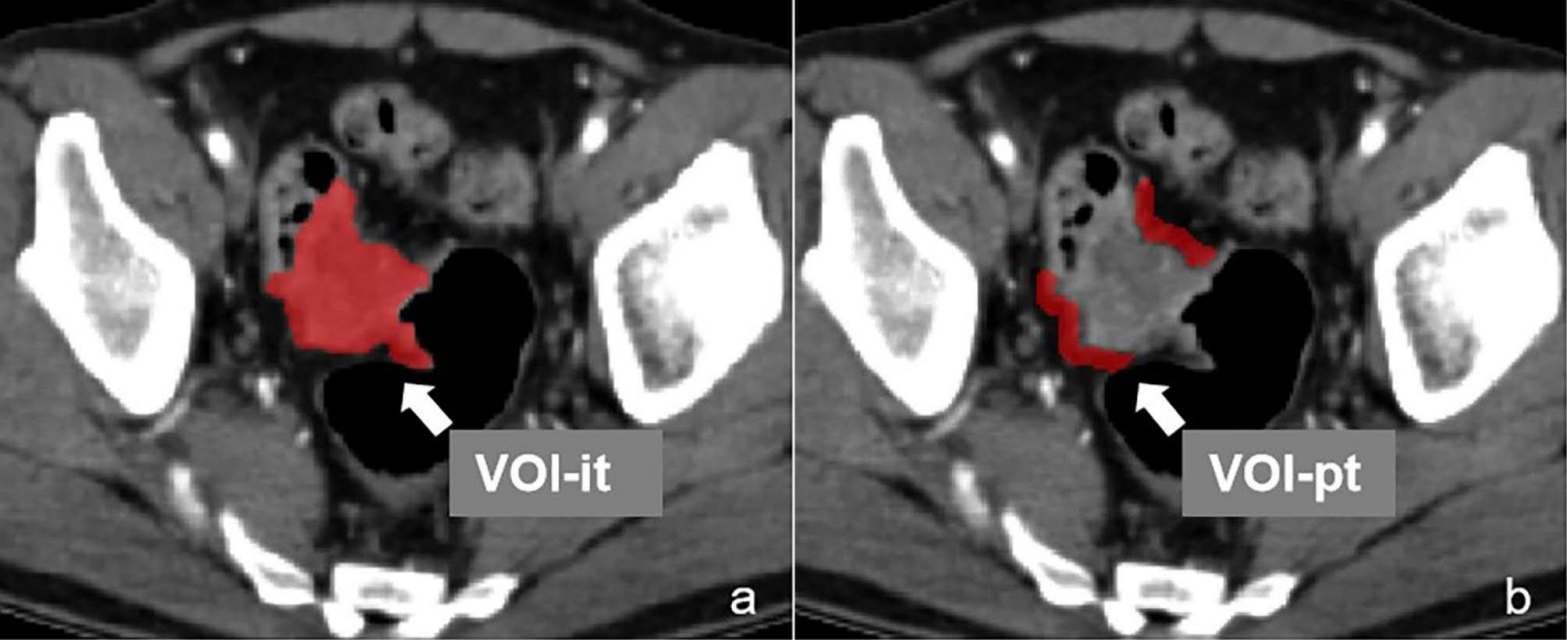
The VOI-it (a) and VOI-pt (b) was delineated in software of ITK-SNAP

Before radiomics analysis, the volume of interest (VOI) of intratumor (VOI-it) and peritumor (VOI-pt) was depicted after three steps: (1) standardize the original CT images through the methods of reconstructing the voxel of X/Y/Z axes into 1.0 mm and adjust the image grayscale into 1 to 32 in software of A.K. (Artificial Intelligence Kit, GE Healthcare). (2) load the standardized triphasic CT images into ITK-SNAP software (https://www.itksnap.org/, Version3.4.0 ), the VOI-it (Fig. 2a) was segmented manually by two radiologists with 7 and 10 diagnostic experience. (3) the VOI-pt (Fig. 2b) was obtained by expanding 5 mm from the margin of tumor in A.K. software.

After segmentation of VOI, the radiomics features of intratumoral and peritumoral tissue were calculated in A.K. software, automatically. Then the repeatability of VOI between two radiologists were evaluated by the analysis of intra-observer correlation coefficient (ICC) among all of the recruited 788 patients. The radiomics features larger than 0.75 were selected and the mean values of selected radiomics features between two radiologists were taken for further analysis. After that, four steps were put into effect to screen radiomics features: (1) the cohort of 788 patients was randomly assigned into two groups of the training group (551 patients) and the validation group (237 patients) with a proportionate of 7:3. (2)before analyses, variables with zero variance were excluded, the outlier values were replaced by the median, and the data were standardized by standardization. (3) the approaches of variance, correlation analysis, and gradient boosting decision tree (GBDT) were employed to extract radiomics features. The specific information of segmentation and radiomics analysis was listed in **Supplementary Material**.

In the end, the five machine learning radiomics models of Bayes, k-nearest neighbor (KNN), logistic regression (LR), support vector machine (SVM), and decision tree (DT) were constructed. The relative standard deviation (RSD) of 100 Bootstrap replication in the training group was calculated, and the machine learning radiomics model with the minimal RSD value showed the higher stability of the model was selected for further analysis [17]. The equation and detail results of RSD were listed in **Supplementary Material**. Then the intratumoral and peritumoral combined machine learning model was conducted. Ten-fold cross-validation was performed in the training group to select the best diagnostic classifier. The Delong test was used to depicted the receiver operator curve (ROC) and the area under curve (AUC) with 95% confidence interval (CI) was calculated to evaluate the efficacy of the model.

### Statistical analysis

The general clinical characteristics including gender, age, long diameter, location, PNI, EMVI, MSI, CEA, CA19-9, history of diabetes, hypertension, smoking, and drinking were analyzed in SPSS software (Version 22). The continuous variables conforming to normal distribution were analyzed by a method of independent t-test, and the categorical variables were analyzed by chi-square test. The methods of radiomics analysis including variance, correlation analysis, GBDT, machine learning algorithms, and logistic-based nomogram were proceeded in R software (Version 3.4.1) and Python (Version 3.5.6). The methods of ICC and ROC were analyzed in MedCalc software (Version 18.2.1). A two-tailed *p*-value < 0.05 indicated a statistical significance.

## Results

### General clinical characteristics

There were 788 RC patients enrolled and the general clinical characteristics were listed in Table 1. The clinical characteristics of gender, age, long diameter, location, MSI, history of diabetes, hypertension, smoking, and drinking. There were 63 low RCs, 114 medium RCs, and 126 high RCs with LNM. The mean age of RCs with LNM was 62.95 ± 11.72 years old and the mean long diameter was 3.92 ± 1.35 cm. There were statistical significance in clinical variables of lesion long diameter (*p* = 0.048), PNI (*p* = 0.000), EMVI (*p* = 0.000), CEA (*p* = 0.034), and CA19-9 (*p* = 0.002). The RCs with LNM had the higher values of CEA (48.97 ± 350.00 µg/L vs. 6.23 ± 12.11 µg/L) and CA19-9 (54.18 ± 177.44 U/mL vs. 20.86 ± 85.92 U/mL) compared with RCs without LNM.

**Table 1:** General clinical characteristics

	Training cohort (n = 551)		Validation cohort (n = 237)		*P*-value
	**LNM**	**non-LNM**	**LNM**	**non-LNM**	
Gender					0.662
Male (%)	135(24.50%)	226(41.02%)	54(22.78%)	84(35.44%)	
female (%)	77(13.97%)	113(20.51%)	37(15.61%)	62(26.16%)	
Age	63.18 ± 10.61	63.13 ± 11.45	62.42 ± 14.03	64.55 ± 12.02	0.481
Diameter (mean ± SD, cm)	3.95 ± 1.44	3.77 ± 1.58	3.85 ± 1.10	3.58 ± 1.45	0.048
Location					0.262
low (%)	46(8.35%)	80(14.52%)	17(7.11%)	38(16.03%)	
medium (%)	80(14.52%)	143(25.95%)	34(14.35%)	49(20.68%)	
high (%)	86(15.61%)	116(21.05%)	40(16.88%)	59(24.89%)	
PNI (%)	93(16.88%)	71(12.89%)	36(15.19%)	34(14.35%)	0.000
EMVI (%)	152(27.59%)	69(12.52%)	67(28.27%)	41(17.30%)	0.000
MSI (%)	27(4.90%)	47(8.53%)	10(4.22%)	13(5.49%)	0.947
CEA (mean ± SD,µg/L)	60.17 ± 416.98	6.62 ± 12.92	22.87 ± 49.47	5.35 ± 9.98	0.034
CA19−9 (mean ± SD,U/mL)	61.86 ± 205.40	20.20 ± 90.44	36.30 ± 79.22	22.40 ± 74.61	0.002
Diabetes (%)	22(3.99%)	41(7.44%)	14(5.91%)	15(6.33%)	0.887
Hypertension (%)	67(12.16%)	135(24.50%)	36(15.19%)	44(18.57%)	0.406
Smoking (%)	40(7.26%)	73(13.25%)	17(7.17%)	25(10.55%)	0.632
Drinking (%)	27(4.90%)	58(10.53%)	11(4.64%)	24(10.13%)	0.097

### Radiomics-based machine learning analysis

**Fig. 3:**
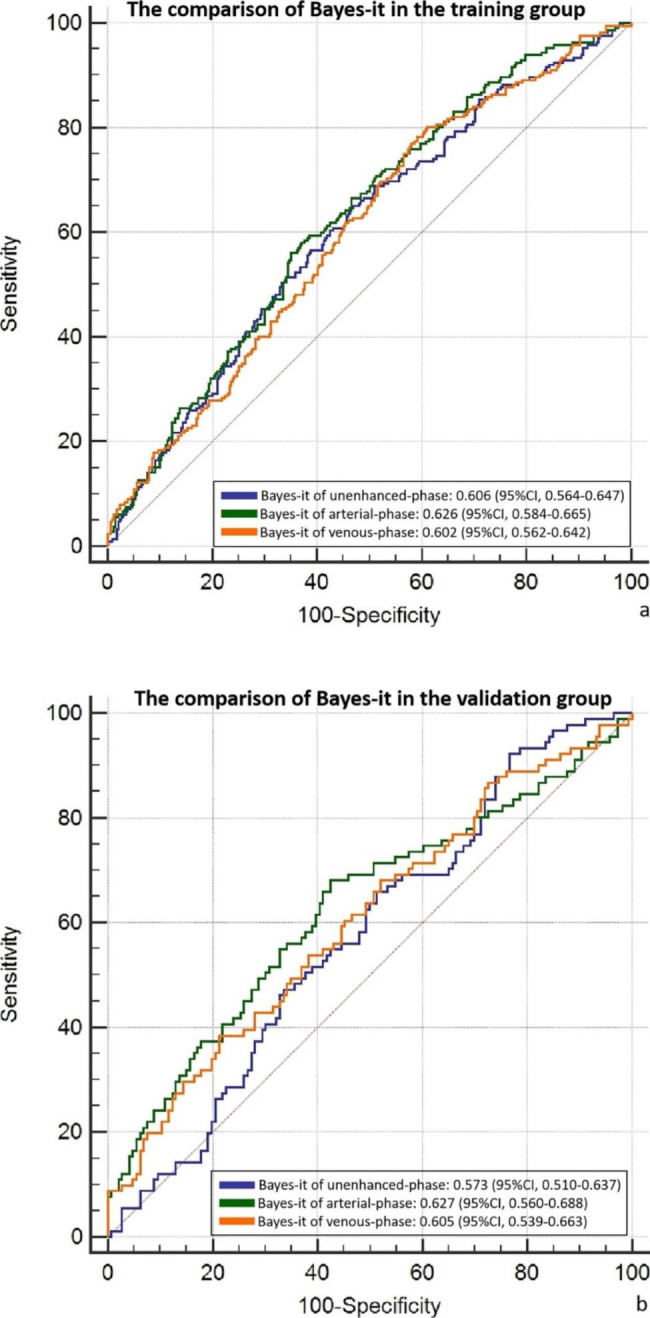
The comparison of machine learning algorithm of Bayes based on intratumoral radiomics in the training group (a) and validation group (b)

To the machine learning of intratumoral radiomics, the RSD values of Bayes machine learning models of triphasic CT images to evaluate the status of LNM were 2.6818%, 2.6754%, and 2.4462%, which were the lowest compared with these of KNN, LR, SVM, and DT. Therefore, the machine learning algorithm of Bayes was chosen to develop models in predict the status of LNM. After comparing the AUCs (Fig. 3a,b) of Bayes models of unenhanced-phase, arterial-phase, and venous-phase, the Bayes model of arterial-phase appeared the considerable prediction the LNM status of RCs (0.626 vs. 0.606 and 0.602 in the training group, 0.627 vs. 0.573 and 0.605 in the validation group), though there was no significance difference after Delong test. Hence, the arterial-phase based intratumoral (Bayes-it) and peritumoral (Bayes-pt) machine learning models of Bayes algorithm were developed for predict the LNM status of RCs. There were 396 intratumoral and 396 peritumoral radiomic features included, and 345 features remained after the method of Variance, then 93 featuers remained after the method of correlation analysis, and 36 features left after the method of GBDT. To the peritumoral radiomics machine learning analysis, the AUCs of Bayes-pt were 0.641 (95%CI, 0.602–0.680) in the training group and 0.617 (95%CI, 0.557–0.677) in the validation group. The specific comparison of Bayes-it of unenhanced-phase, arterial-phase, and venous-phase by Delong test was listed in the **supplementary material**.

### Clinical-Bayes nomogram construction

**Fig. 4:**
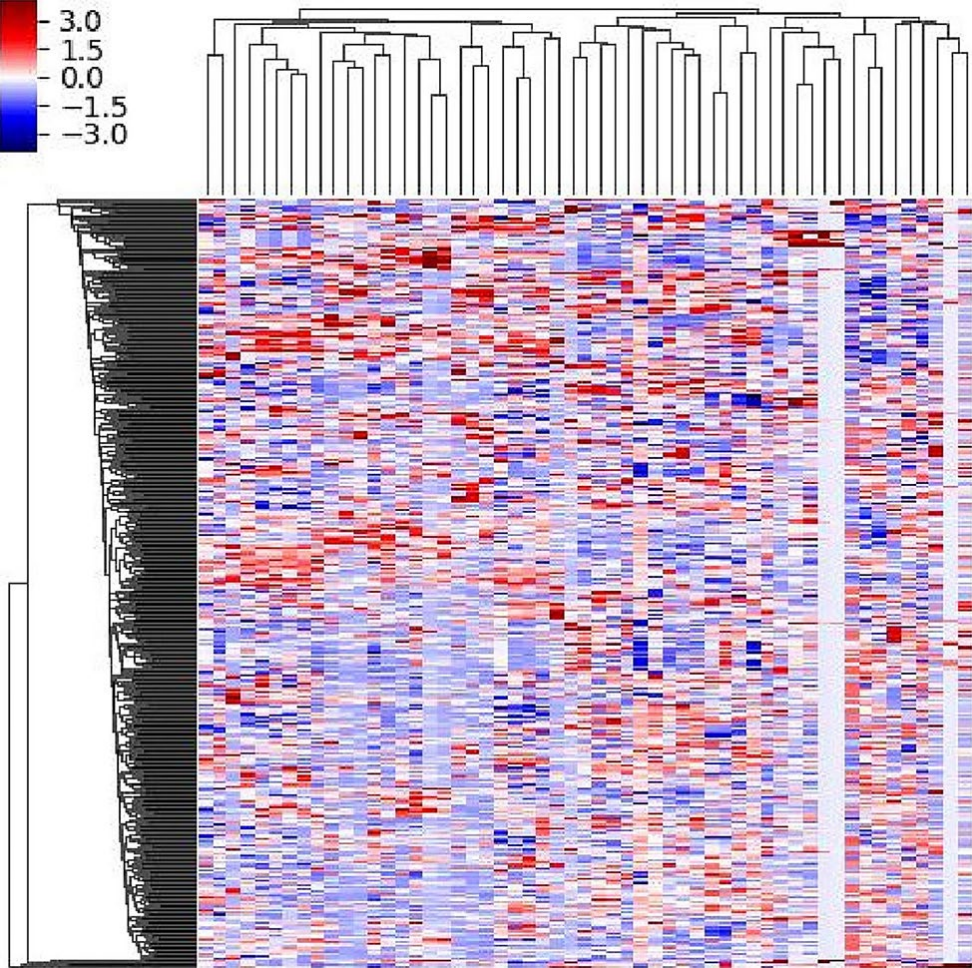
The heatmap of intratumoral and peritumoral radiomics in the training group after GBDT method

The Bayes machine learning model combined intratumoral and peritumoral radiomics (Bayes-it/pt) was constructed, including 23 intratumoral radiomics features and 32 peritumoral radiomics features after GBDT method to select features. The heatmap of intratumoral and peritumoral radiomics in the training group after GBDT method was illustrated in Fig. 4. The heatmap displayed the selected radioimc features in this data matrix, and the color changes were used to visualize and compare their correlation. The AUCs of Bayes-it/pt were 0.656 (95%CI, 0.616–0.692) in the training group and 0.638 (95%CI, 0.574–0.698) in the validation group. And the corresponding Bayes score (Bayes-score) was quantified.

**Fig. 5:**
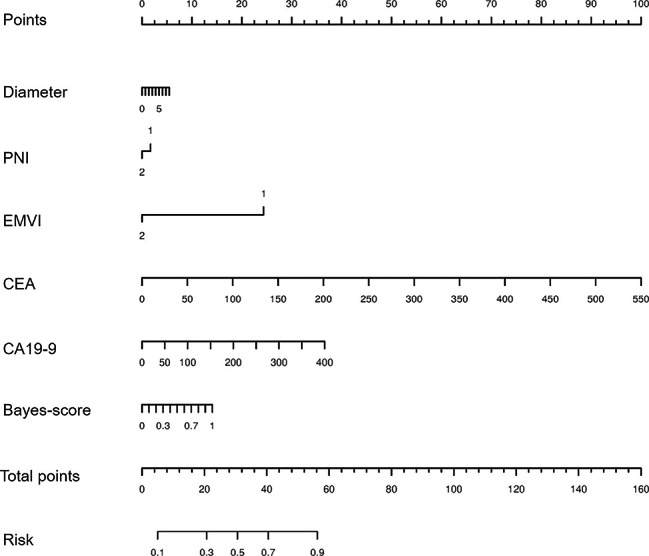
The clinical-Bayes nomogram including Bayes-score, diameter, PNI, EMVI, CEA, and CA19-9 to predict the LNM status of RCs

Then, the clinical-Bayes nomogram including Bayes-score, diameter, PNI, EMVI, CEA, and CA19-9 was developed to predict the LNM status of RCs (Fig. 5). The clinical-Bayes nomogram showed the best performance with AUC of 0.828 (95%CI, 0.800-0.854), sensitivity of 77.23%, and specificity of 74.85%. The calibration curve listed in the **Supplementary Material** and non-significant Hosmer-Lemeshow test (*p* = 0.719) showed the goodness-of-fit of this nomogram.

## Discussion

Our study focused on the radiomics-based machine learning to predict the LNM status of RCs. To compare the prediction stability of different machine learning algorithms, we used the indicator of RSD and the model with the minimal RSD value was considered to be the most stable one. The results showed that the machine learning algorithm of Bayes had the minimal RSD value in all of unenhanced-phase, arterial-phase, and venous-phase machine learning models. Therefore ,the Bayes algorithm was chosen for next step analysis. And the AUCs of Bayes-it model of arterial-phase were slightly higher than these of unenhanced-phase and venous-phase models (0.626 vs. 0.606 and 0.602 in the training group, 0.627 vs. 0.573 and 0.605 in the validation group), though there were no statistical significance by Delong test. So we selected the machine learning algorithm of Bayes in arterial-phase to further predict the LNM status of RCs. As has been previously investigated that multi-objective radiomics based on T2WI images helped to predict preoperative LNM status of RCs [18]. According to our study, the AUCs of Bayes-it/pt were around 0.65, whose diagnostic performance is not particular good, but it still could provide auxiliary information beyond the basis of conventional CT characteristics.

The overestimation of LNM may lead to unnecessary neoadjuvant therapy, resulting in potential complications such as impaired continence function and so on [19]. On the contrary, the underestimation of LNM will lead to the absence of preoperative neoadjuvant chemoradiotherapy, which will increase the recurrence and metastatic rate [20]. Therefore, accurate preoperative prediction of lymph nodes is helpful for the determination of optimal treatment. Conventional CT images evaluated the LNM of RCs based on the size and morphological of lymph nodes, suggesting that the possibility of malignancy should be warned if the lymph node greater than 4.5 mm in diameter, though this criterion has not been widely accepted [21]. The radiomics nomogram including radiomics, CT-reported lymph node status, and CEA showed good discrimination of the LNM status of colorectal carcinoma [22]. Our intratumoral and peritumoral radiomics-based Bayes machine learning analysis showed that simple intratumoral and peritumoral radiomics showed similar AUCs in predicting LNM status of RCs (0.626 and 0.641 in the training group, 0.627 and 0.617 in the validation group). Therefore the combined intratumoral and peritumoral Bayes radiomics was analyzed, with the higher AUCs of 0.656 (95%CI, 0.616–0.692) and 0.638 (95%CI, 0.574–0.698) in both the training and validation group compared with single ones. But the single CT-based radiomics analysis to predict the LNM status of RC is still not satisfactory.

Moreover, in order to improve the predictive efficacy, the significant clinical factors of diameter, PNI, EMVI, CEA, and CA19-9 were taken into account. The clinical-Bayes nomogram including Bayes-score and these clinical factors was developed, with the AUC, specificity, and sensitivity of 0.828 (95%CI, 0.800-0.854), 74.85%, and 77.23%. Additionally, the combination of clinical, histological, and MRI-based intratumoral radiomics has been reported to predict the LNM status in breast cancer [23], prostate cancer [24], and so on. Therefore the detection of clinical-Bayes nomogram contained intratumoral and peritumoral radiomics, clinical factors of diameter, PNI, EMVI, CEA, and CA19-9 was tremendously significant for preoperative detecting LNM of RCs with the highest AUC compared with model of Bayes-it, Bayes-pt, and Bayes-it/pt.

There were several limitations in this article. First, this retrospective study included the RC with the pathology of signet-ring cell carcinoma and mucinous carcinoma for the reason to comprehensively analyze different types of RC. While, the signet-ring cell carcinoma and mucinous carcinoma had a significant different biological behavior and prognosis from classical adenocarcinoma [25], the further study about the distinction between them is needed. Second, due to the irregular shape of RCs, the bias between manual segmentation may affect the radiomic analysis, though the ICCs were calculated to reduce the intra-observer difference. An automatic approach to segment the RCs for radiomic analysis needed to be further explored. Third, regarding this single-center design, a multi-center validation is necessary to identify the performance of this model.

## Conclusion

Intratumoral and peritumoral radiomics based Bayes analysis helped to predict the LNM status of RCs. And the clinical-Bayes nomogram containing Bayes-score, and significant clinical variables of diameter, PNI, EMVI, CEA, and CA19-9 showed a considerable superiority over predicting the LNM status of RCs.

## Electronic supplementary material

Below is the link to the electronic supplementary material.

Supplementary Material 1

## Data Availability

The datasets used and analyzed in this article is available from the corresponding author on reasonable request. The code used in this study is available at GitHub (https://github.com/mayq1988/).
