# Supplementary material for: The CT-based intratumoral and peritumoral machine learning radiomics analysis in predicting lymph node metastasis in rectal carcinoma
Source: BMC Gastroenterol. 2022 Nov 16;22:463. doi: 10.1186/s12876-022-02525-1 (PMC9670407; doi:10.1186/s12876-022-02525-1)
Supplement: Supplementary file 1 — Supplementary Material 1 [file 12876_2022_2525_MOESM1_ESM.doc]

**Supplementary Material**

1. **Radiomics features**

The radiomics features includes histogram parameters, texture parameters, Form Factor parameter, Gray level co-occurrence matric (GLCM) parameters, gray level run-length matric (GLRLM) parameters, and gray level Size Zone Matric (GLZSM) parameters.

**Histogram parameters:** It concerns with properties of individual pixels. They describe the distribution of voxel intensities within the CT image through commonly used and basic metrics. It covers the followed parameters: Energy, Entropy, Max-Intensity, Min-Intensity, Mean Value, Mean absolute deviation, Median-Intensity, Range ,Root mean square (RMS), Standard deviation (std Deviation), Uniformity, Variance, Volume Count, Voxel Value Sum, Relative Deviation, Frequency Size, Quantiles, Percentiles, Skewness, and Kurtosis.

**Texture parameters:** It is one of the important characteristics used in identifying objects or regions of interest in an image, texture represents the appearance of the surface and how its elements are distributed. It is considered an important concept in machine vision, in a sense it assists in predicting the feeling of the surface (e.g. smoothness, coarseness, and so on) from image. It covers the followed parameters: Energy, Entropy, Correlation, Inertia, Cluster Shade, Cluster Prominence.

**Form Factor parameters:** It includes descriptors of the three-dimensional size and shape of the tumor region. It covered the followed parameters: Sphericity, Surface area, Compactness 1, Compactness 2, Inertia, Cluster Shade, Cluster Prominence.

**GLCM parameters:** It represents the joint probability of certain sets of pixels having certain gray level values. It calculates how many times a pixel with gray level i occurs jointly with another pixel having a gray value j. By varying the displacement vector d between each pair of pixels. It covers the followed parameters: Energy of GLCM, Entropy of GLCM, Inertia of GLCM, Correlation, Inverse Difference Moment, Haralick features. And the haralick features includes Haralick Correlation, Angular Second Moment, Contrast, Haralick Entropy, Hara Variance, sum Average, sum Variance, sum Entropy, difference Variance, difference Entropy, inverse Difference Moment.

**GLRLM parameters:** It is defined as the numbers of runs with pixels of gray level i and run length j for a given direction θ. RLMs is generated for each sample image segment having directions (0°, 45°, 90°&135°). It covers the followed parameters: Short/Long Run Emphasis, Gray Level Non-uniformity, Run Length Non-uniformity, Low/High Gray Level Run Emphasis, Short Run Low/High Gray Level Emphasis, Long Run Low/High Gray Level Emphasis.

**GLZSM parameters:** It is the starting point of Thibault matrices. For a texture image f with N gray levels, it is denoted GSf (s, g) and provides a statistical representation by the estimation of a bi-variate conditional probability density function of the image distribution values. It covers the followed parameters: Small/Large Zone Emphasis, Gray-level Non-uniformity, Zone-Size Non-uniformity, Zone Percentage, Low/High Gray-Level Zone Emphasis, Small Zone Low/High Gray-Level Emphasis, Large Zone Low/High Gray-Level Emphasis, Gray-Level Variance, Zone-Size Variance.

1. **The preprocess of radiomics signature**

The preprocess of radiomics signature comprises the following steps: Image standardization, VOI segmentation, and intraclass agreement (ICC) analysis.

**Image standardization:** We resampled all the images into a 1.0mm*1.0mm*1.0mm voxel size at X/Y/Z-spacing. Then denoising by Gaussian and normalizing image gray level to a scale from 1 to 32 were automatically proceed in software of AK (GE Healthcare).

**VOI segmentation:** 3D volume of intratumoral interest (VOI-it) were delineated by two radiologists with 7 and 10 years of experience independently, in ITK-SNAP software. The manual defined smooth curve VOI was delineated the tumor margin. Then the peritumoral VOIs (VOI-pt) were automatically obtained after expanding 5mm from the contour of tumor in the software of AK. The regions of necrosis, intraluminal air, non-invaded rectal wall, vessel, and peri-rectal fat were eliminated from contours of VOIs, manually. Finally, the intratumoral and peritumoral radiomic features were calculated on A.K. software, automatically.

**The intraclass agreement (ICC) analysis:** The intra-observer agreement of feature extraction were evaluated by the value of ICC. Intra-observer ICC was computed by comparing extractions of reader A (with 7 years’ experience on abdominal CT) and reader B (with 10 years’ experience on abdominal CT). When the ICC was greater than 0.75, it was considered as good agreement and favorable extraction reproducibility. And the mean value of radiomics features from two radiologists were calculated as robust features for further analysis.

1. **Radiomic feature selection**

The cohort of 788 patients was randomly assigned into two cohorts of the training cohort (551 patients) and the validation cohort (237 patients) with a proportionate of 7:3. Before analyses, variables with zero variance were excluded from analyses. Then, the outlier values were replaced by the median. Finally, the data were standardized by the standardization. Standardization: Extracted texture features were standardized, which removed the unit limits of the data of each feature and converted it into a dimensionless pure value. This allowed the indexes of different units or orders to be compared and weighted. We used a z-score normalization to make the image intensities fit a standard normal distribution with$\mu\text{=0}$and$\sigma\text{=1}$, where$\mu$is the mean value of the images, and$\sigma$is the standard deviation. The normalized values (also called z-scores) of the image intensities (*x*) were calculated as follow:

$$z=\frac{x-\mu}{\sigma}$$

The process of feature selection included univariate analysis and multivariate logistic analysis with stepwise selection method. Firstly, analysis of variance on the extracted features was performed. The variance value is the average of the square of the difference between the value of each variable and the mean. It is the most important method for measuring the dispersion of numeric data. The larger the variance, the greater the fluctuation of the data, and vice versa. So, it is necessary to preferentially eliminate features with a variance of 0 or less. In this study, the variance of each feature was calculated, and then the features greater than the threshold 1 were retained. Second, the correlation test was calculated to reduce data redundancy. The software calculated the paired correlation between each two of the features. If the correlation coefficient was greater than 0.7, which showed that the two features were highly correlated, one of them was removed. Third, the method of gradient boosting decision tree (GBDT) was used to select radiomic features. Gradient boosting decision tree (GBDT) is a Boosting algorithm based on decision tree as base learner. It builds a decision tree in each iteration to reduce the residual of the current model in the gradient direction. GBDT is commonly used for regression, classification and feature selection. GBDT’s advantages include: (a) It flexible processes of various types of data, including both continuous and discrete data set; (b) It has powerful predictive ability and generalization ability; (c) It has good interpretability, and robustness, can automatically discover high-order relationships between features, and does not require data normalization and other processing^[1]^.

1. **The prediction of machine learning models**

The indicator of relative standard deviation (RSD) of 100 Bootstrap replication in the training cohort was used to predict the stability of all machine learning models. The RSD with minimal value showed the higher stability. The equation of RSD was:
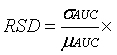
100 %, σAUC and µAUC are the standard deviation and mean of the 100 AUC values from 100 Bootstrap replication in the training set, respectively. The RSD values of Bayes, k-nearest neighbor (KNN), logistic regression (LR), support vector machine (SVM), and decision tree (DT) of triphasic CT images were calculated.

The RSD values of unenhanced-phase, arterial-phase, and venous-phase CT images of Bayes, KNN, LR, SVM, and DT intratumoral machine learning models in the training cohort:

|  | Bayes | KNN | LR | SVM | DT |
| --- | --- | --- | --- | --- | --- |
| Unenhanced-phase | 2.6818 | 2.8399 | 3.0235 | 5.3379 | 6.3414 |
| Arterial-phase | 2.6754 | 2.9640 | 2.7273 | 4.1167 | 5.5164 |
| Venous-phase | 2.4462 | 2.7767 | 2.8271 | 3.9871 | 5.6733 |

The RSD values of Bayes in the unenhanced-phase, arterial-phase, and venous-phase were the minimal, and the machine learning algorithm of Bayes was selected for further analysis.

The Delong test was utilized to compare the ROC curves of unenhanced-phase, arterial-phase, and venous-phase in the training group and validation group.

| Comparison of triphasic phase of Bayes-it | Training group (*p)* | Validation group (*p)* |
| --- | --- | --- |
| Unenhanced-phase vs. Arterial-phase | 0.569 | 0.341 |
| Unenhanced-phase vs. Venous-phase | 0.917 | 0.579 |
| Arterial-phase vs. Venous-phase | 0.478 | 0.659 |

The comparison of ROC curves of Bayes machine learning models in unenhanced-phase, arterial-phase, and venous-phase in the training and validation cohort:


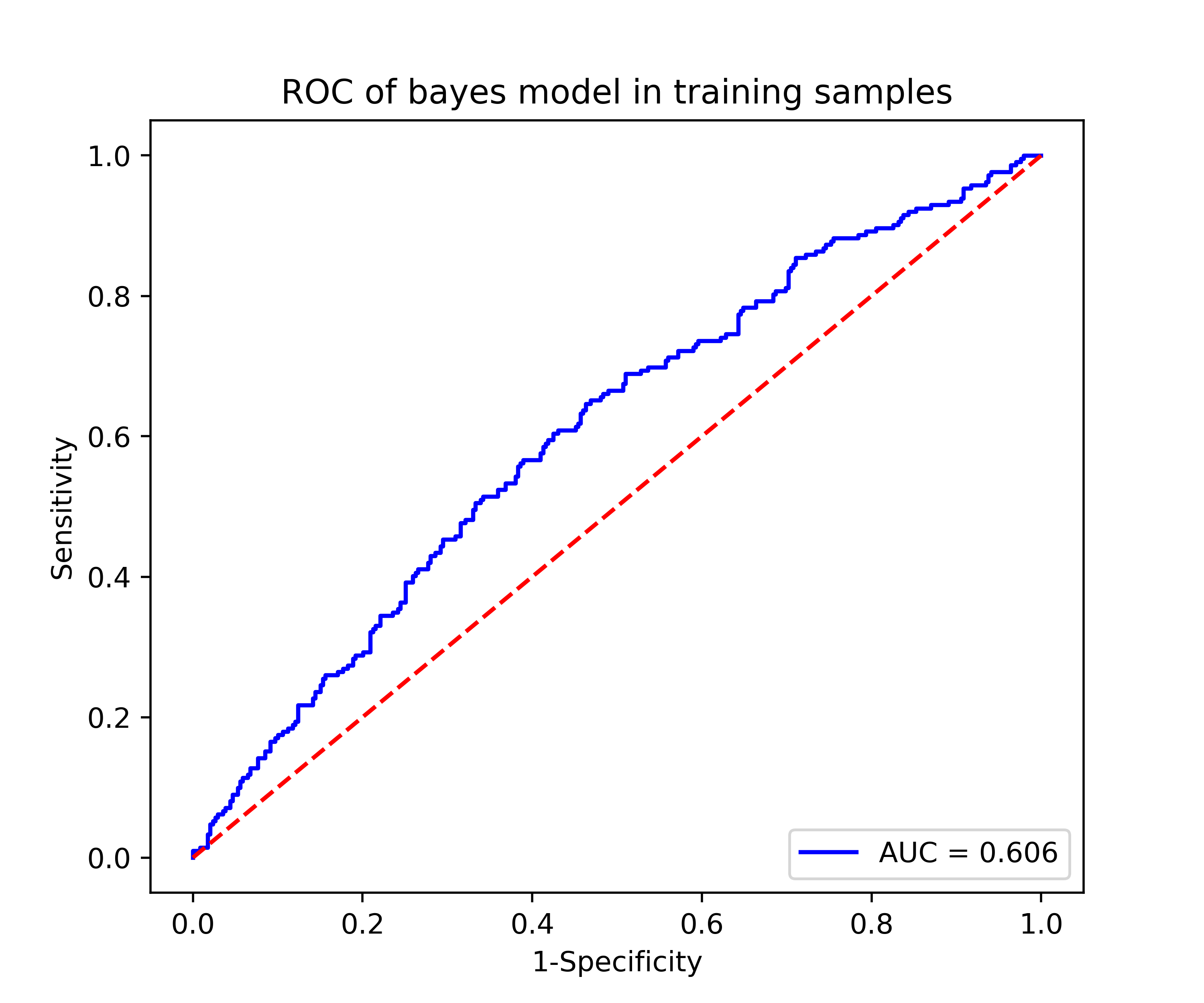

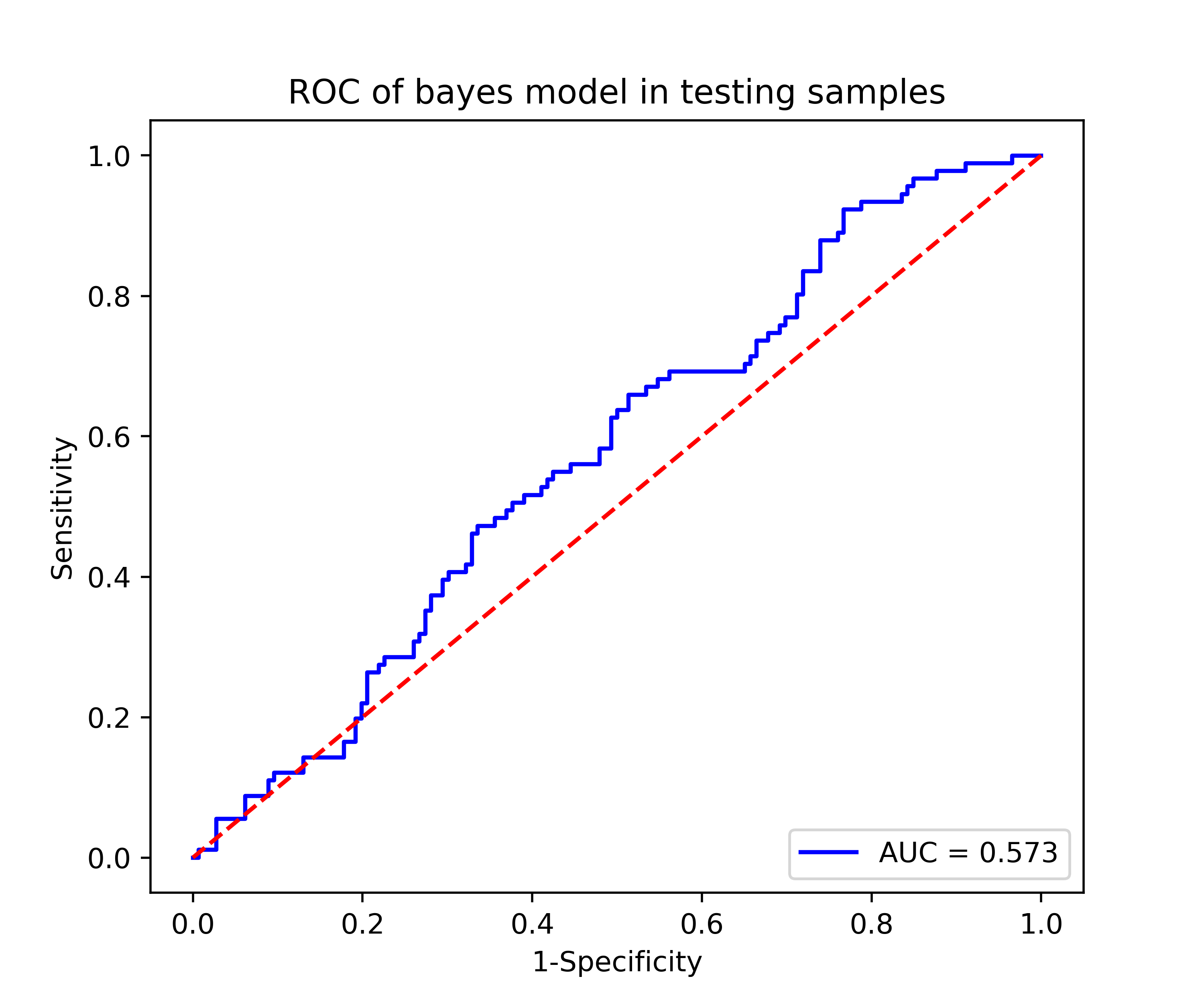


**Unenhanced-phase**


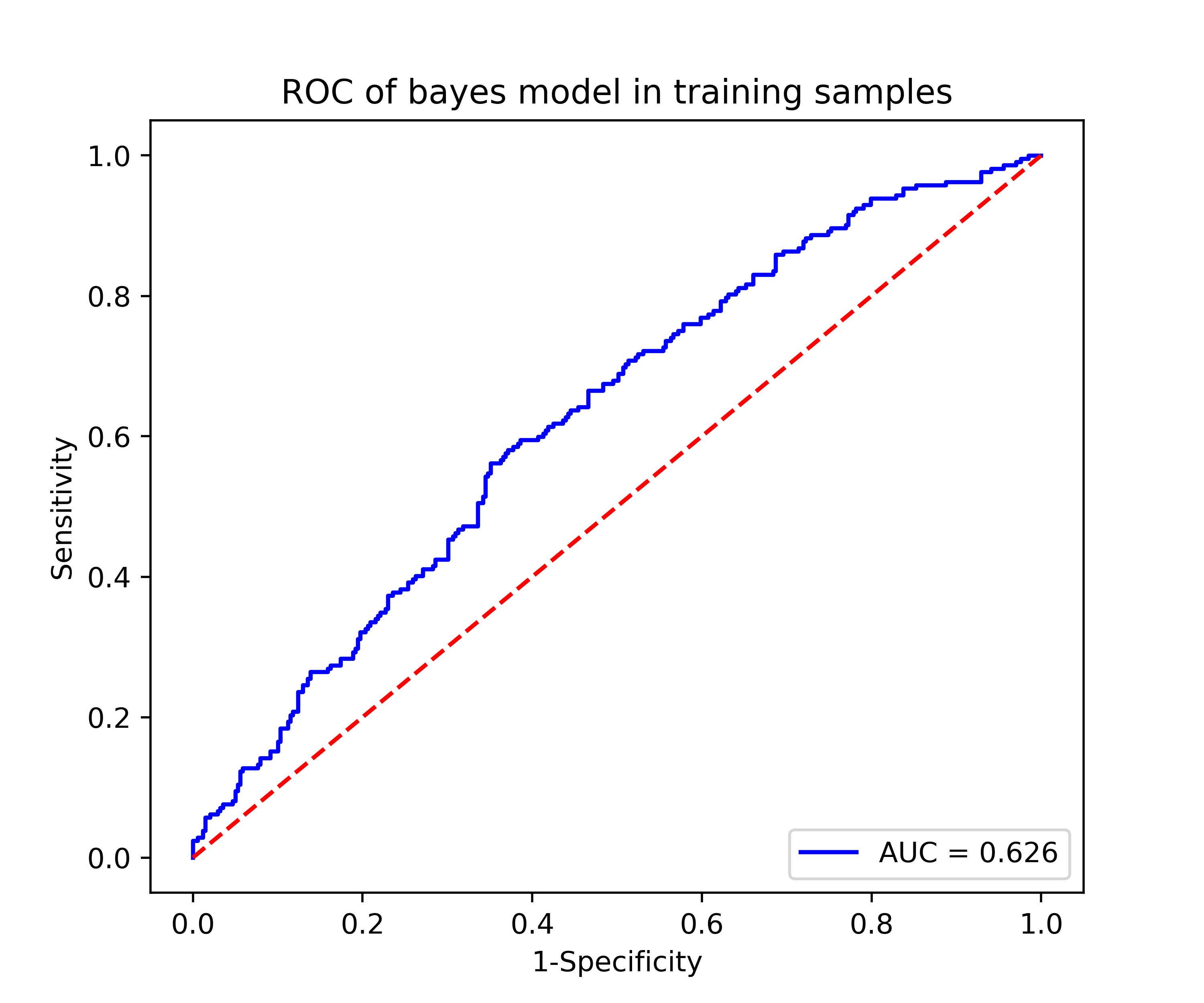

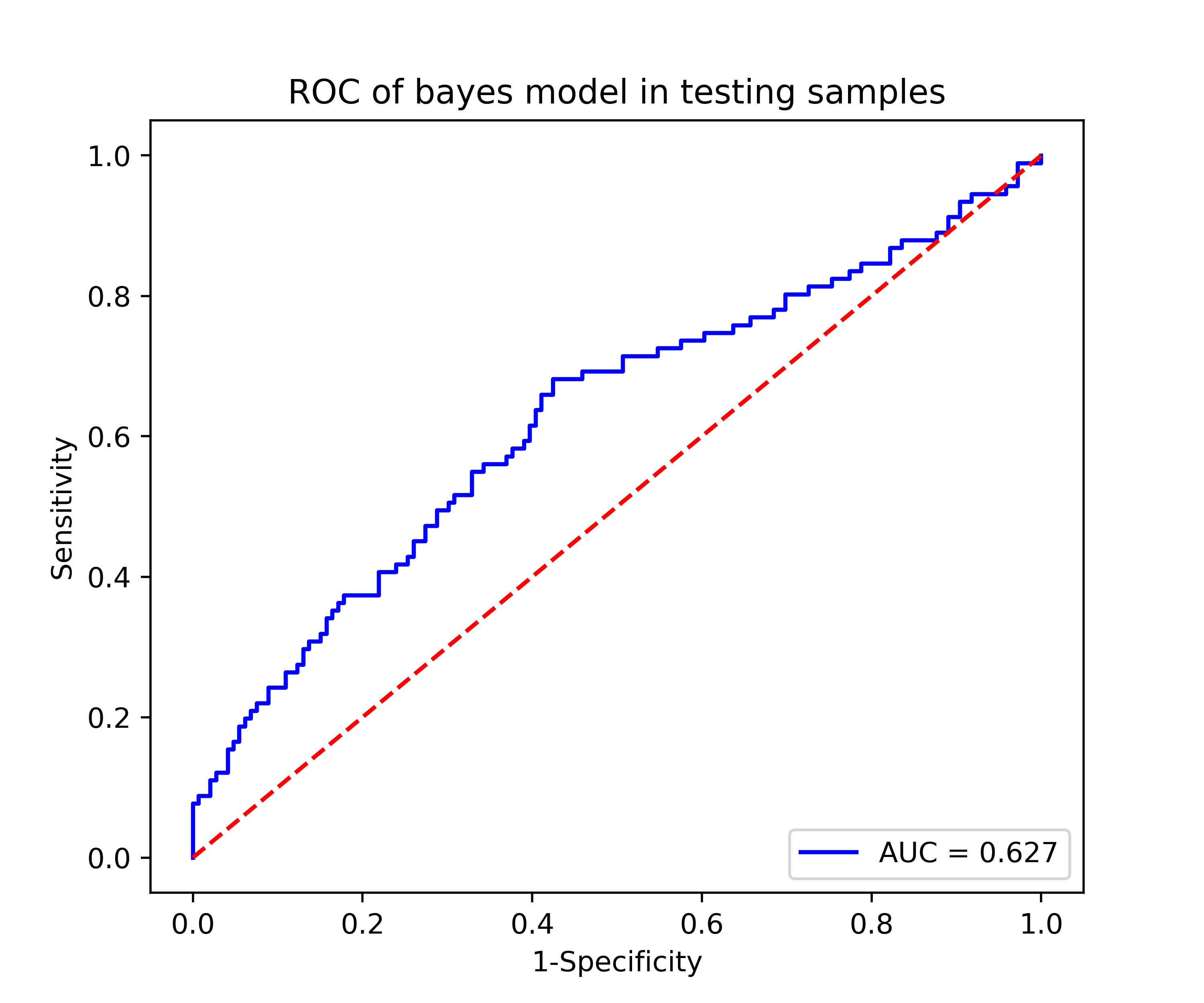


**Arterial-phase**


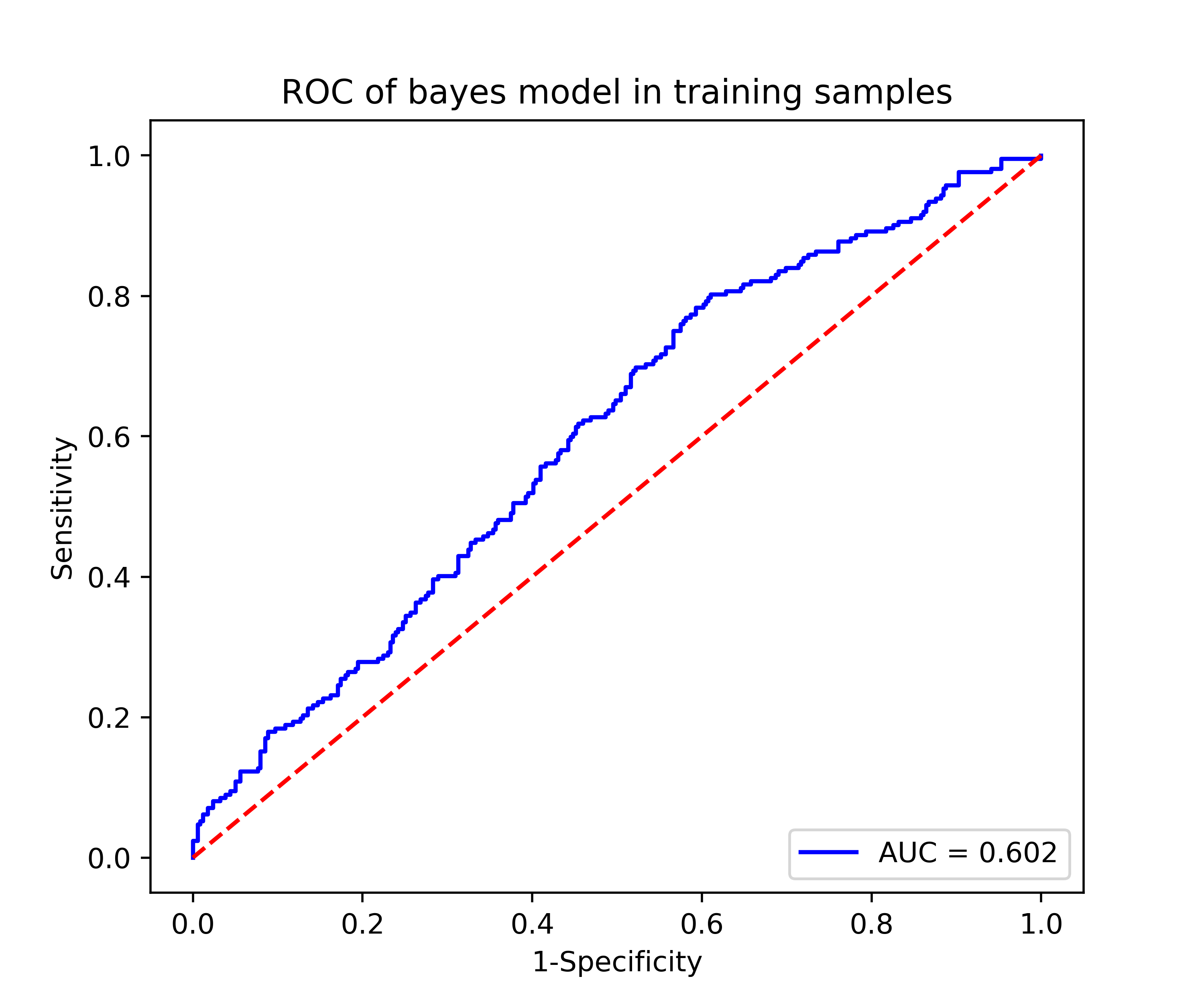

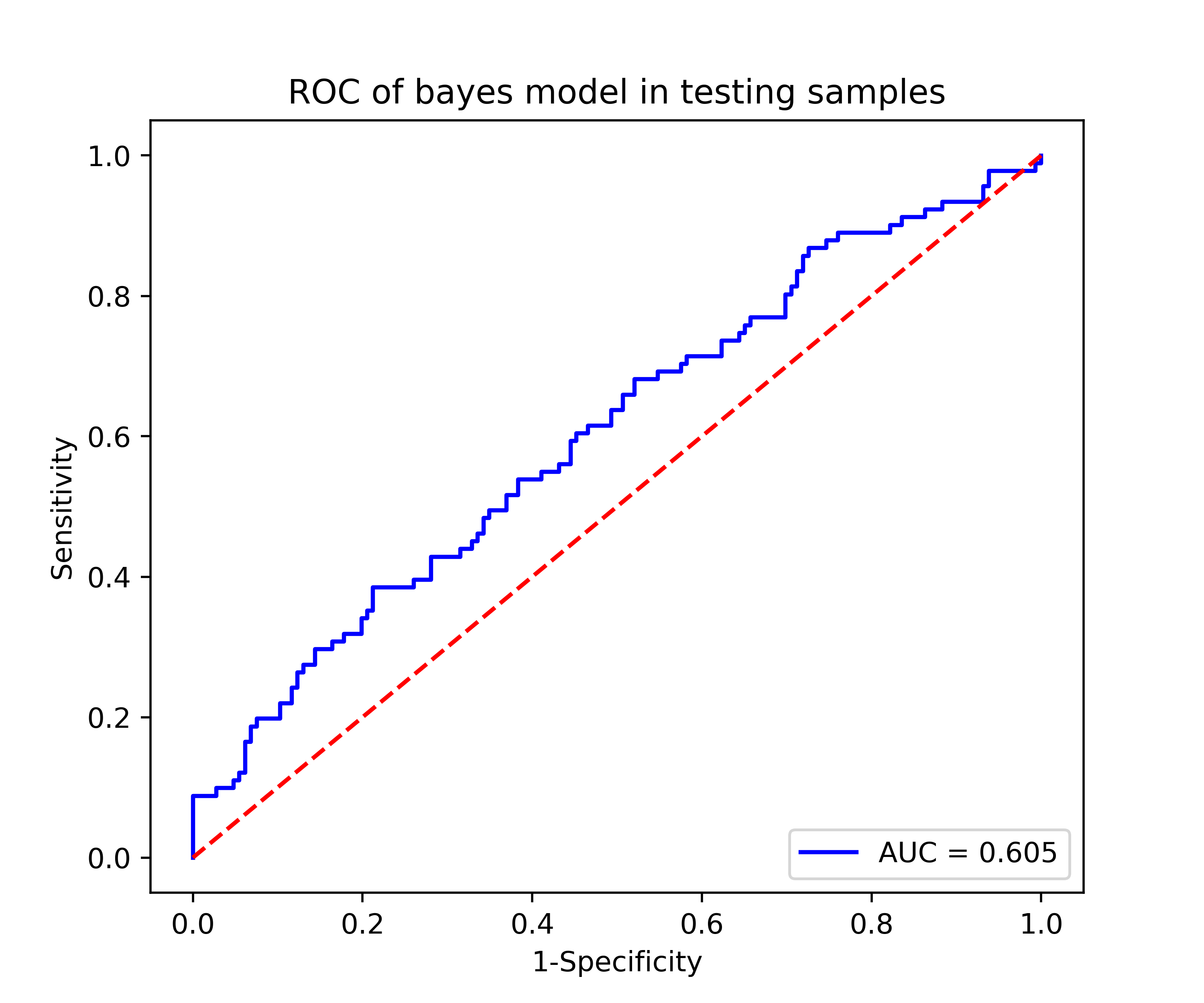


**Venous-phase**

Though, there were no statistical difference between the triphasic phase of Bayes-it, the AUCs of arterial-phase was slightly higher than those of unenhanced-phase and venous-phase in both the training group and validation group. Therefore, the arterial-phase was chosen for further analysis.

1. **The intratumoral and peritumoral Bayes machine learning models**


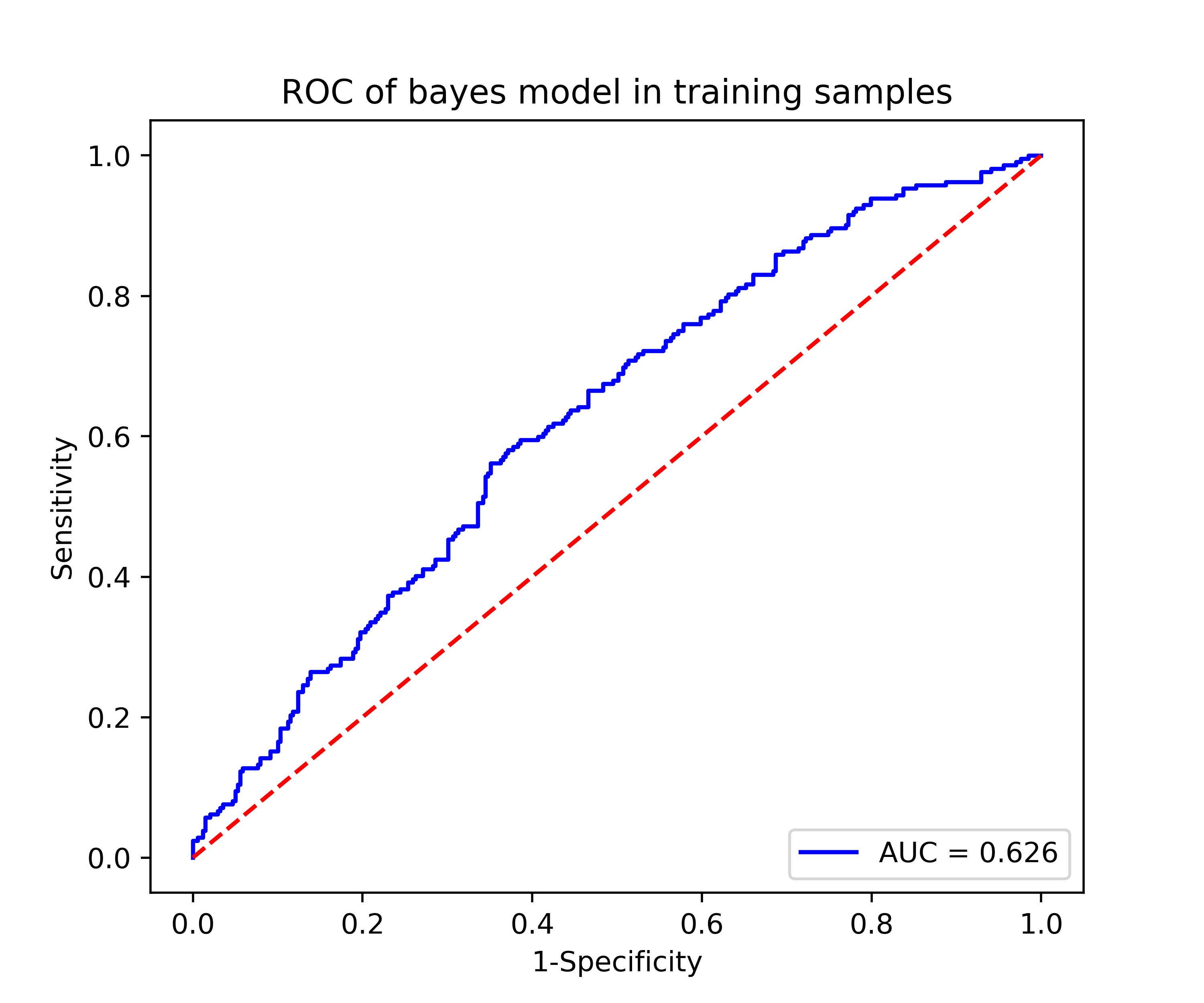

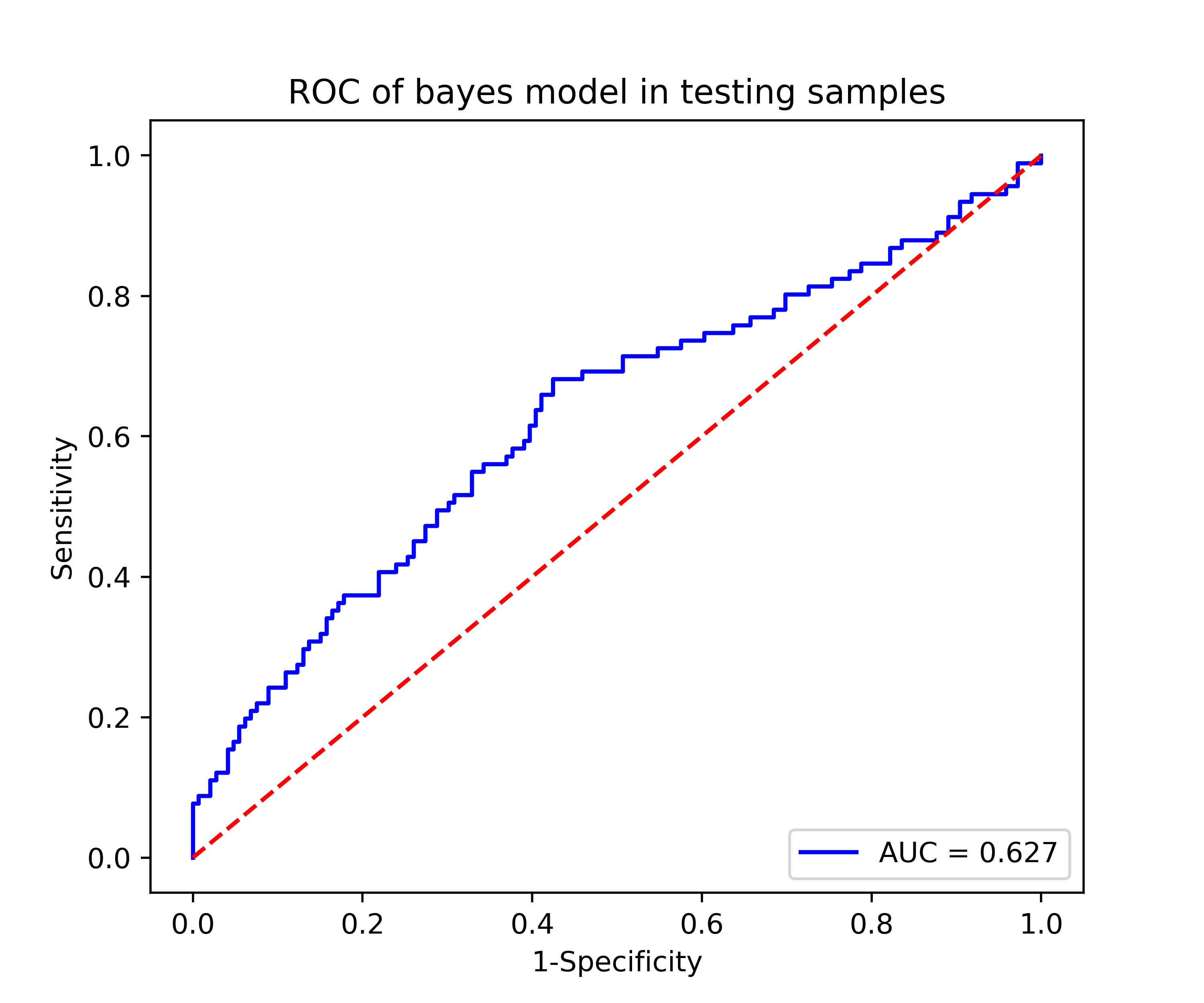


The ROC curves of Bayes-it with the AUC of 0.626 in the training group and 0.627 in the validation group.


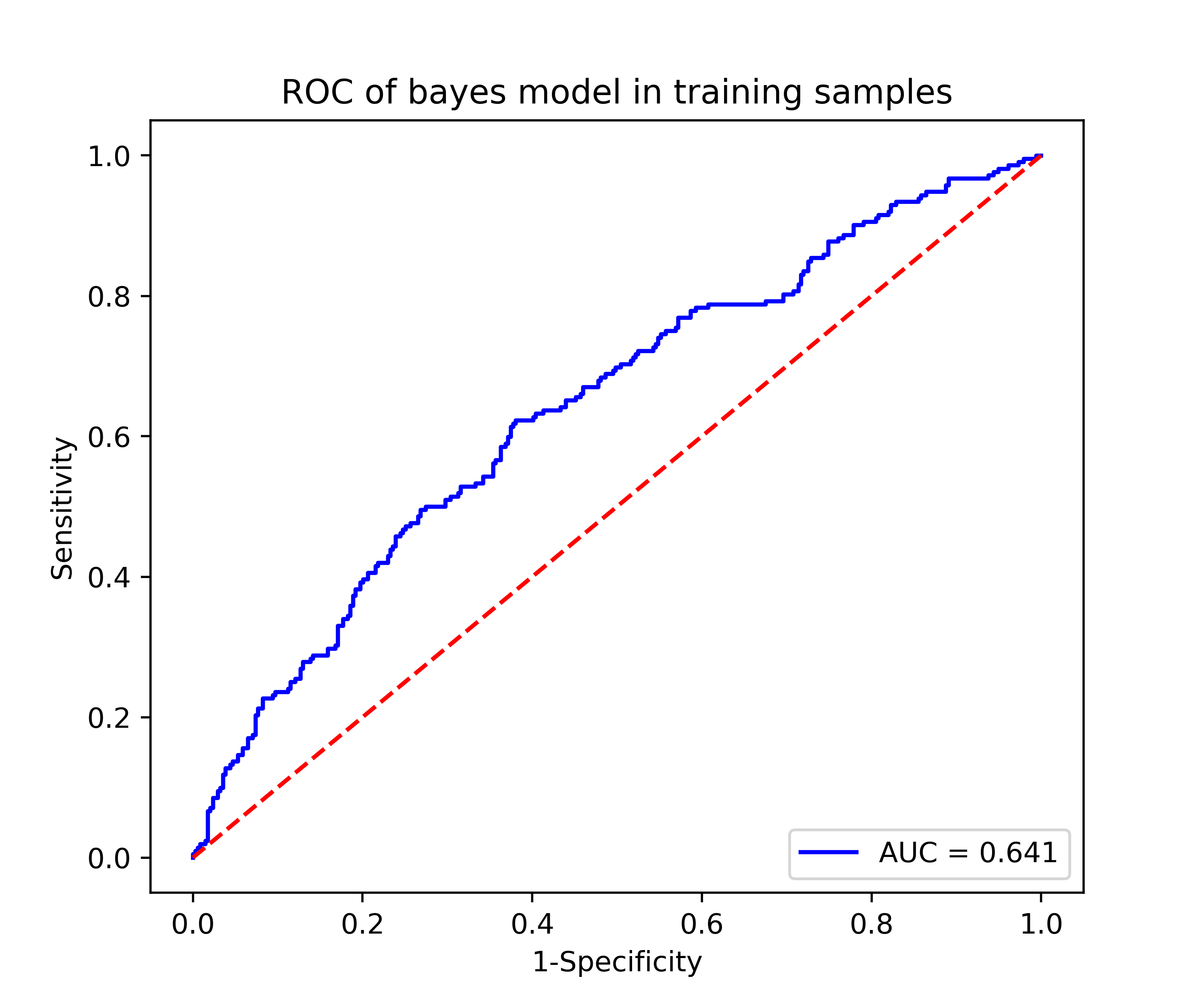

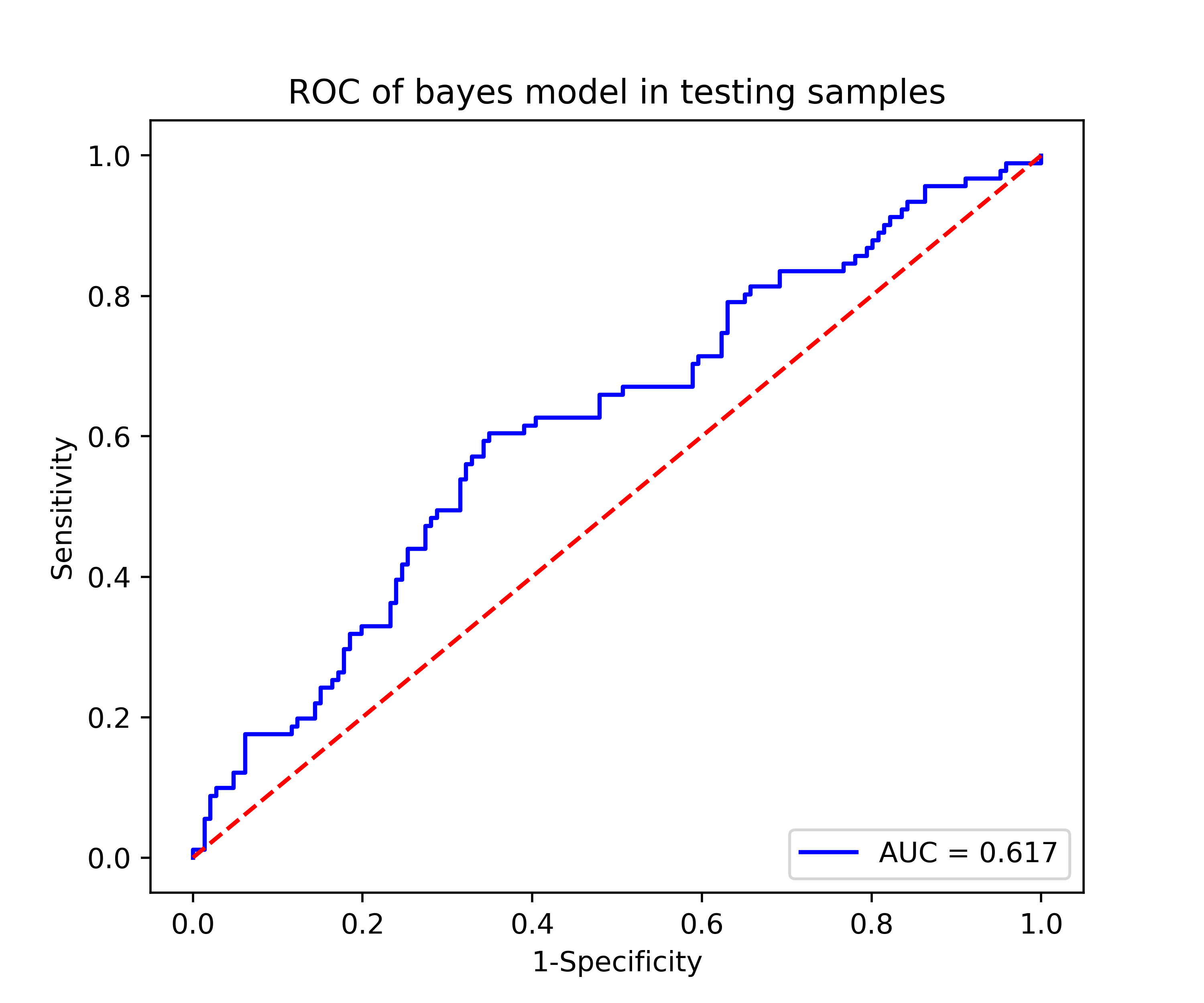


The ROC curves of Bayes-pt with the AUC of 0.641 in the training group and 0.617 in the validation group.


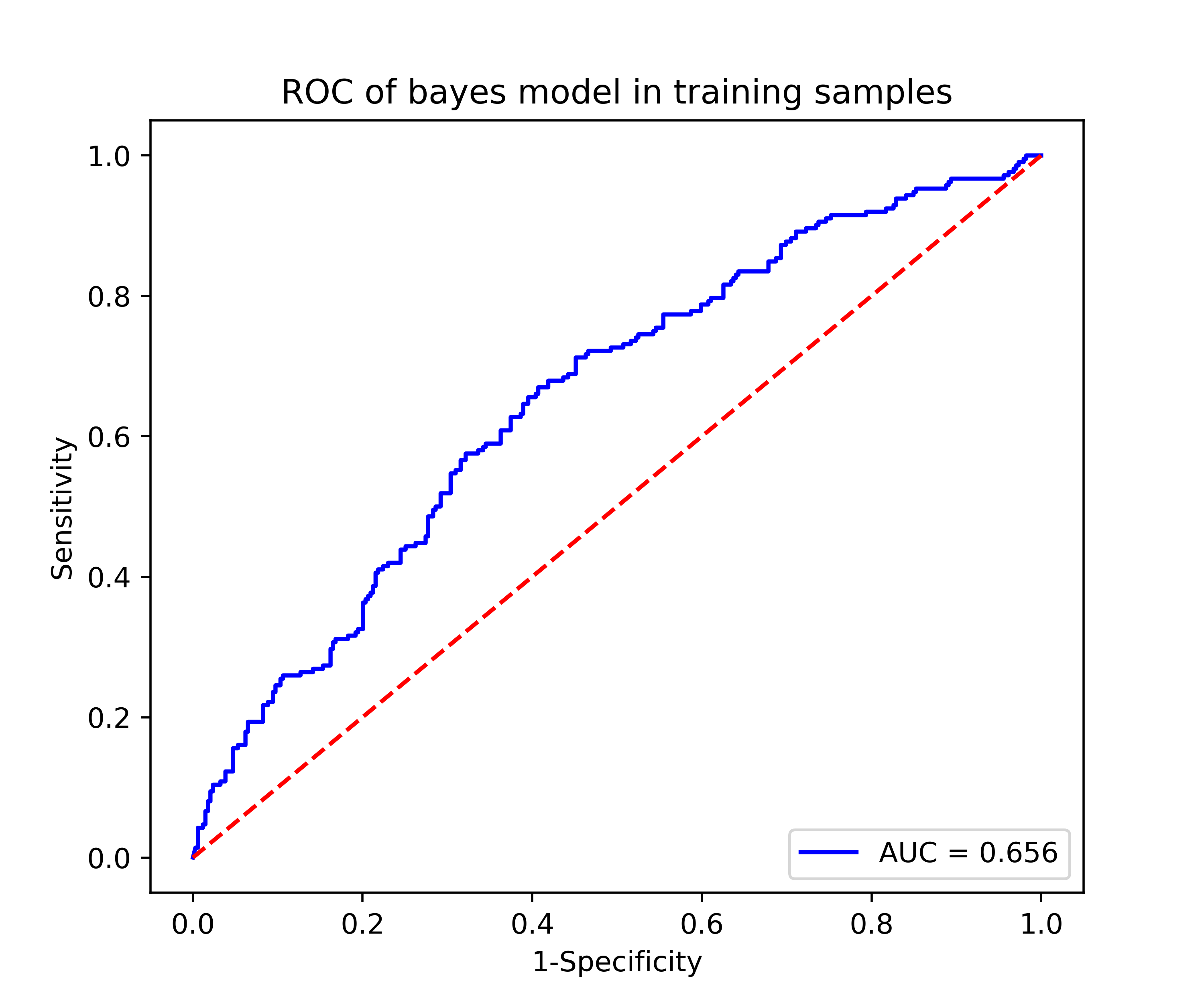

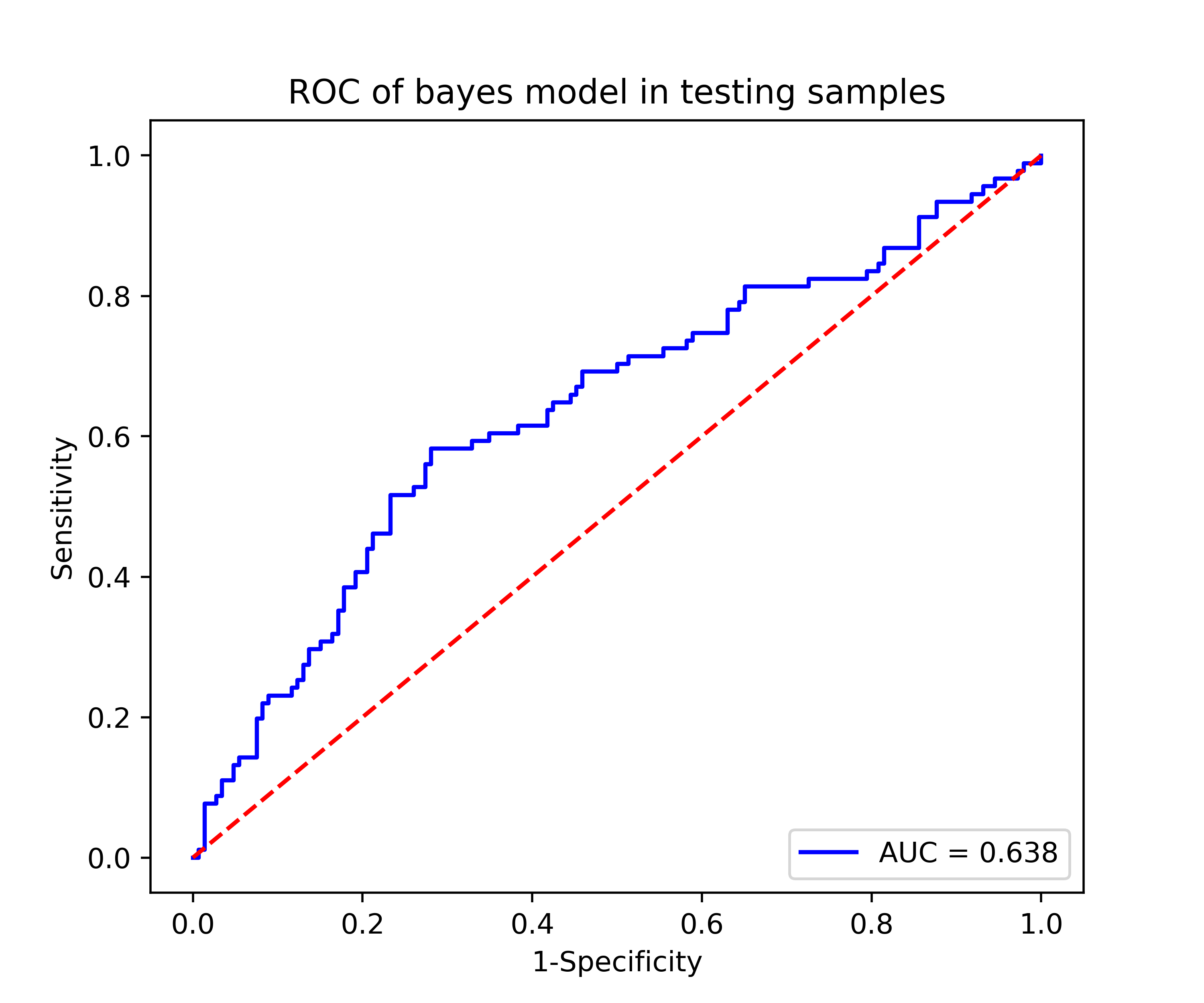


The ROC curves of Bayes-it/pt with the AUC of 0.656 in the training group and 0.638 in the validation group.

The corresponding scores of Bayes-it/pt (Bayes-score) of the training group and validation group were as below:


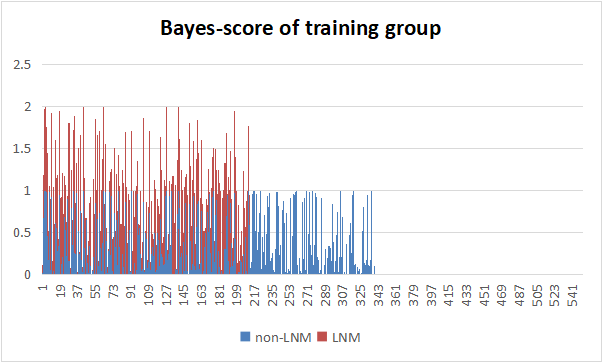


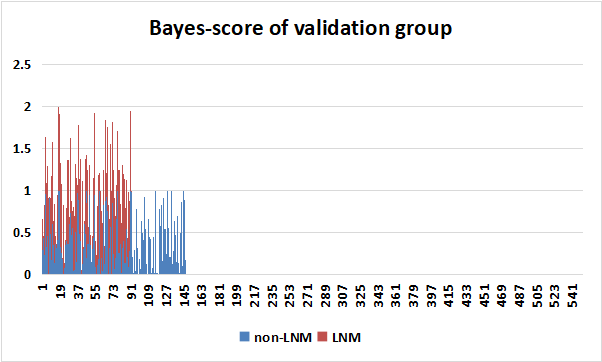


1. **The construction of clinical-Bayes nomogram**

The clinical-Bayes nomogram was developed including variables of diameter, PNI, EMVI, CEA, CA19-9, and Bayes-score. The calibration curves were as below:


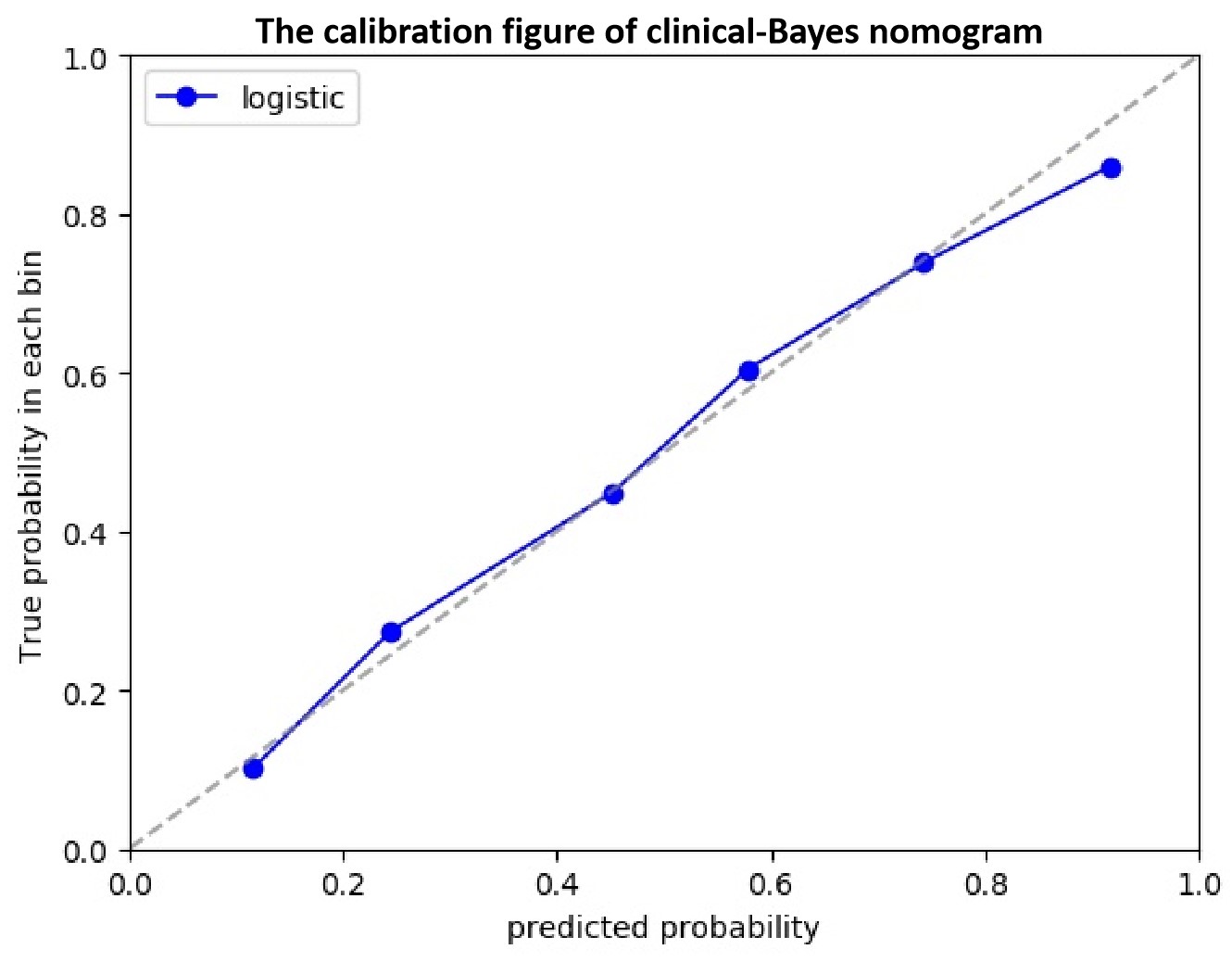


1. **Reference**

Liang Y, Zhang S, Qiao H, Cheng Y. iEnhancer-MFGBDT: Identifying enhancers and their strength by fusing multiple features and gradient boosting decision tree. Math Biosci Eng. 2021 Oct 14;18(6):8797-8814. doi: 10.3934/mbe.2021434. PMID: 34814323.
